# Impact of One-Week Administration of Dihydrotestosterone in Rat Anterior Pituitary Gland

**DOI:** 10.1155/2022/9525227

**Published:** 2022-10-21

**Authors:** Haruhiko Kanasaki, Tuvshintugs Tumurbaatar, Zhouma Cairang, Zolzaya Tumurgan, Aki Oride, Hiroe Okada, Satoru Kyo

**Affiliations:** Department of Obstetrics and Gynecology, Shimane University School of Medicine, Izumo 693-8501, Japan

## Abstract

Hyperandrogenism causes dysfunction of the hypothalamic–pituitary–gonadal (HPG) axis in reproductive women. In this study, we examined the effects of dihydrotestosterone (DHT) on characteristic changes in rat anterior pituitary gland samples. DHT was administered to ovary-intact 6-week postnatal female rats for 7 days, after which the anterior pituitary glands were examined and compared with those in control rats. Estrous cyclicity was not drastically disrupted by DHT treatment. Common gonadotropin *α* subunit (*Cga*), luteinizing hormone *β* subunit (*Lhb*), and follicle-stimulating hormone (FSH) *β* subunit (*Fshb*) gene expression levels were not modulated by DHT treatment, while prolactin (*Prl*) gene expression was significantly repressed by DHT. Gonadotropin-releasing hormone (GnRH) receptor (*Gnrh-r*) gene expression was significantly inhibited by DHT, whereas pituitary adenylate cyclase-activating polypeptide (PACAP) receptor (*Pca1-r*) gene expression was increased by DHT. Gene expression levels of the receptors encoded by thyrotropin-releasing hormone (*Trh-r*) and kisspeptin (*Kiss1-r*) genes were unchanged. Expression of inhibin *α* subunit (*Inha*) and activin *β*A subunits (*Actba*) within the pituitary was inhibited by DHT treatment, while activin B subunit (*Actbb*) and follistatin (*Fst*) gene expression was unchanged by DHT. In mouse pituitary gonadotroph L*β*T2 cells, DHT did not modulate the gene expression of *Gnrh-r*, but it inhibited the expression of *Inha* and *Actba* subunits within the L*β*T2 cells. In rat prolactin-producing GH3 cells, DHT did not modulate prolactin gene expression, but it increased *Pac1-r* gene expression. The present observations suggest that DHT directly or indirectly affects the anterior pituitary gland and induces characteristic changes in hormone-producing cells.

## 1. Introduction

Excess androgen levels disrupt the hypothalamic–pituitary–gonadal (HPG) axis and induce reproductive dysfunction. Hyperandrogenism is one of the medical conditions characterized by higher levels of serum androgens, and its clinical symptoms include acne, obesity, increased body or facial hair, and the induction of irregular or absent menstruation in women [1]. Hyperandrogenism in women can be caused by various conditions, including congenital adrenal hyperplasia, ovarian hyperthecosis, and androgen-producing tumors or drugs [2]. In particular, polycystic ovary syndrome (PCOS), which affects up to 20% of reproductive-age women [3], could account for a considerable proportion of cases of hyperandrogenism [4]. At present, women are diagnosed with PCOS if they exhibit two out of the three following features: clinical and/or biochemical hyperandrogenism, anovulation, and polycystic ovary morphology as revealed by ultrasound [5].

Excessive androgen exposure induces the development of PCOS-like traits in primates, sheep, and rodents [6–8]. Genetic modification of the androgen receptor to complete receptor insufficiency protects against the development of PCOS in mice [9]. Furthermore, it has been reported that blockage of androgen action restores menstrual regularity in some women with PCOS [10].

Elevated serum levels of luteinizing hormone (LH), which is reflected by higher GnRH pulsatility, is one of the endocrinological traits of hyperandrogenism [11]. In addition, a link between hyperprolactinemia and hyperandrogenism has been proposed in patients with PCOS [12]. Although it remains unknown whether hyperprolactinemia is associated with the pathogenesis of PCOS, hyperprolactinemia and menstrual cycle irregularity in women with PCOS might both be explained by a common hypothalamic-pituitary abnormality. A previous study indicated that high levels of LH in women with PCOS might be caused by decreased dopaminergic tone, which is responsible for negatively regulating prolactin release [13]. Of course, there are conflicting results regarding the effect of dopamine inhibitors on LH levels in PCOS women [14]. Another hypothesis suggests hyperandrogenism causes hyperprolactinemia because PCOS induces hyperestrogenemia [15]. Indeed, oestrogen can increase the secretion of prolactin [16].

Female reproductive function is controlled by the HPG axis, and dysfunction of this axis is involved in the anovulatory endocrine status of patients with hyperandrogenism. At present, kisspeptin neurons in the infundibular nucleus of the hypothalamus that govern the pulsatile secretion of GnRH and kisspeptin neurons in this area are hypothesized to play a pivotal role in sex−steroid–induced negative feedback control in women [17]. Because increases in the serum levels of LH are reflected by higher GnRH pulsatility, several studies have focused on kisspeptin neurons and androgens to elucidate the pathogenesis of PCOS [18, 19]. However, it is possible that hyperandrogenemia directly affects the pituitary level and modulates the secretion of pituitary hormones.

In this study, an animal model of hyperandrogenemia was developed using ovary-intact female rats, and the changes in expression levels of gonadotropin subunits and prolactin genes were determined. In addition, receptors for hypothalamic peptides and inhibin subunits expressed in the anterior pituitary gland were examined after DHT treatment. Furthermore, confirmatory experiments using pituitary hormone-secreting cell models were performed.

## 2. Materials and Methods

### 2.1. Materials

The following chemicals and reagents were obtained from the indicated sources: GIBCO foetal bovine serum (Invitrogen, Carlsbad, CA); dihydrotestosterone (DHT), penicillin-streptomycin and thyrotropin-releasing hormone (TRH) (Sigma-Aldrich Co., St. Louis, MO); Activin A and inhibin A (Abcam, Cambridge, MA).

### 2.2. In Vivo Experiments using Ovary-Intact Female Rats

Six-week-old female Wistar rats (The Jackson Laboratory Japan Inc., Yokohama, Japan) were maintained under a 12 h light/dark cycle at 20°C–25°C with food (CE-2; CLEA Japan, Tokyo, Japan) and water available *ad libitum*. Rats were housed two per cage. Vaginal smears are easily obtained from rats at this age, and smears were assessed daily to evaluate their estrous cyclicity. The rats received a daily subcutaneous injection of DHT (5 mg/kg/day) (*n* = 4) to produce a supraphysiological androgen level *in vivo*, based on a previous study [20], or a placebo control (*n* = 4) in 160 mL of sesame oil (Fujifilm, Tokyo, Japan) for 7 days. Then, the rats were euthanized while under isoflurane anesthesia, and the pituitary gland was removed. Anterior pituitary tissues were extracted and subjected to quantitative RT-PCR analysis. This protocol was approved by the ethics committee of the Experimental Animal Center for Integrated Research at Shimane University (IZ31-51).

#### 2.2.1. Cell Culture and Stimulation

L*β*T2 cells (kindly provided by Dr. P. Mellon of the University of California, San Diego, CA) and GH3 cells (CCL-82.1, American Type Culture Collection) were plated in 35-mm tissue culture dishes and incubated with high-glucose Dulbecco's modified Eagle's medium (Sigma-Aldrich Co.) containing 10% heat-inactivated foetal bovine serum and 1% penicillin-streptomycin at 37°C under a humidified atmosphere of 5% CO_2_ in air. For the stimulation experiments, the medium was changed to high-glucose Dulbecco's modified Eagle's medium containing 1% heat-inactivated foetal bovine serum and 1% penicillin-streptomycin, and the cells were incubated without (control) or with the test reagents (DHT, activin A, inhibin A, and TRH) for 24 h. The concentration and stimulation times of DHT, activin A, and inhibin A were chosen because we already knew that these concentrations and stimulation times cause cultured hypothalamic cells to change their characteristics [21, 22].

### 2.3. RNA Preparation, Reverse Transcription, and Quantitative Real-Time Polymerase Chain Reaction

Total RNA was extracted from anterior pituitary tissue or the cultured cells using TRIzol-LS (Invitrogen). To obtain cDNA, 1.0 *µ*g of total RNA was reverse-transcribed using oligo-dT primers (Promega, Madison, WI) and prepared using a First-Strand cDNA Synthesis Kit (Invitrogen) in reverse transcription buffer. The preparation was supplemented with 10 mM dithiothreitol, 1 mM of each dNTP, and 200 U of RNase inhibitor/human placenta ribonuclease inhibitor (Cat. No. 2310; Takara, Tokyo, Japan) in a final reaction volume of 10 *µ*L. The reaction was incubated at 37°C for 60 min. *Cga*, *Lhb*, and *Fshb* subunits, *Prl*, *Gnrh-r*, *Pac1-r*, *Thr-r*, *Kiss-1r*, inhibin and activin subunits (*Inha*, *Actba*, *Actbb*), and *Fst* mRNA levels were determined by using real-time (RT)-PCR (ABI Prism 7000; Perkin-Elmer Applied Biosystems, Foster City, CA) according to the manufacturer's protocol (User Bulletin No. (2) as well as Universal ProbeLibrary probes and Fast Start Master Mix (Roche Diagnostics, Mannheim, Germany). Primer sequences used for expression assays of the above genes are listed in Table 1. *Gapdh* mRNA was used to normalize the amount of cDNA added per sample. For each set of primers, a no-template control was included. The thermal cycling conditions were as follows: 10 min denaturation at 9°C, followed by 40 cycles of 95°C for 15 s and 60°C for 1 min. Reactions were followed by melting curve analysis (55°C–95°C). To determine PCR efficiency, a 10-fold serial dilution of cDNA was performed as described previously [23]. The PCR conditions were optimized to generate >95% efficiency, and only those reactions with between 95% and 105% efficiency were included in subsequent analyses. Relative differences in cDNA concentrations between the baseline and experimental conditions were calculated using the comparative threshold cycle (Ct) method [24]. For each sample, ΔCt was calculated to normalize expression to the internal control (*Gapdh*) by using the following equation: ΔCt = ΔCt(gene) – Ct(*Gapdh*). To determine differences between the experimental and control conditions, ΔΔCt was calculated as ΔCt(sample) − ΔCt(control). Relative mRNA levels were calculated using the following equation: fold difference = 2^ΔΔCt^.

### 2.4. Statistical Analysis

Experiments using cell cultures were repeated independently at least three times. Two sets of samples were prepared and stimulated by different conditions. These two sets of samples were assayed in duplicate. From four sets of data, mean values were determined. The same experiments were repeated three times, and the final mean ± standard errors of mean (SEM) were determined from three sets of means. Statistical analysis was performed using Student's *t*-test in the experiments comparing the two stimulation groups. One-way analysis of variance (ANOVA) with Bonferroni's posthoc test was conducted to analyze the experiments that determined the effects of two doses of stimulant on target gene expression, and two-way ANOVA was applied to the experiments that tested combined stimulation by two stimulants, respectively. Statistical significance was assessed at a *P* < 0.05 threshold. All analyses were performed using Prism 6.07 software (GraphPad Software, San Diego, CA).

## 3. Results

### 3.1. Effect of DHT Administration on Estrous Cyclicity in Ovary-Intact Female Rats

**Figure 1:**
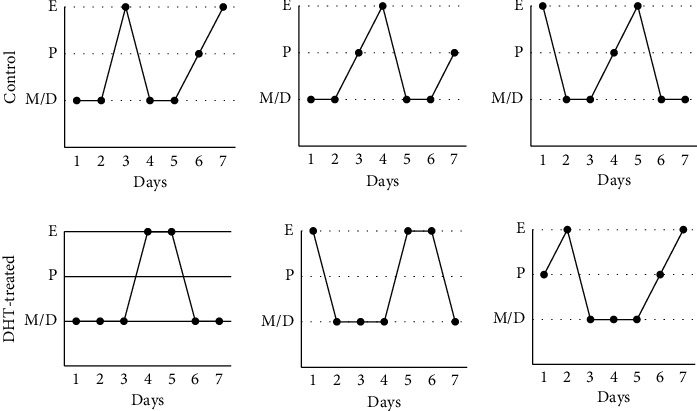
Estrous cyclicity in control and DHT-treated rats. Six-week-old female rats were injected subcutaneously with DHT (5 mg/kg/day) daily. Vaginal smears were collected daily to evaluate estrous cyclicity during treatment. However, DHT-treated rats still showed estrous cyclicity similar to that of control rats. Three representative cycles in control and DHT-treated rats are shown.

DHT was administered for 7 days to ovary-intact female rats, and their estrous cyclicity was monitored by assessing vaginal smears throughout the treatment. During DHT treatment, estrous cyclicity was not drastically disrupted and still showed proestrous and estrus stages (Figure 1).

### 3.2. Effect of DHT Administration on Gonadotropin Subunits and Prolactin Gene Expression within the Anterior Pituitary

**Figure 2:**
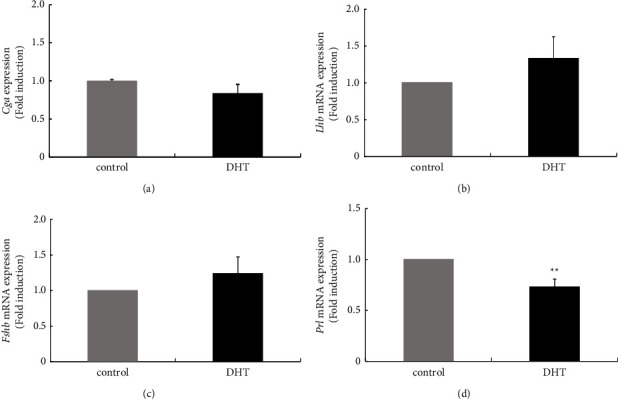
Effect of dihydrotestosterone (DHT) administration on gene expression of pituitary gonadotropin subunits and prolactin. Six-week-old female rats were injected subcutaneously with DHT (5 mg/kg/day) daily for 7 days. After the rats were euthanized, the anterior pituitary was removed from nontreated (*n* = 4) and DHT-treated rats (*n* = 4), and then, mRNA was extracted from the anterior pituitary tissue and reverse transcribed. The mRNA levels of Cga (a), Lhb (b), and Fshb (c) subunits and prolactin were measured by quantitative RT-PCR. Samples from each experimental group were run in duplicate and normalized to the mRNA levels of the housekeeping gene Gapdh. The results are expressed as fold induction over the control and presented as the mean ± SEM. ^*∗∗*^*P* < 0.01 versus control.

To examine the effect of androgen excess on the pituitary gland, DHT was administered for 7 days to ovary-intact female rats. Then, the anterior pituitary gland was removed, and changes in the expression levels of three gonadotropin subunits and prolactin genes were determined. Expression levels of *Cga* and *Lhb* and *Fshb* genes within the anterior pituitary were unchanged by DHT treatment compared with nontreated rats (Figure 2(a)–2(c)). In contrast, *Prl* gene expression in DHT-treated rat pituitary glands was significantly reduced to 0.73 ± 0.07-fold compared with that in nontreated rats (Figure 2(d)).

### 3.3. Effect of DHT Administration on Receptor Gene Expression Levels for Hypothalamic Peptides in the Anterior Pituitary

**Figure 3:**
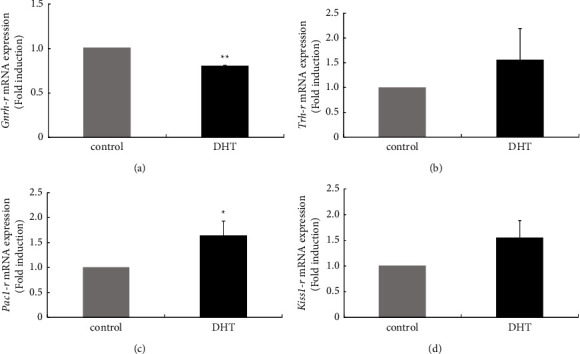
Effect of dihydrotestosterone (DHT) administration on receptor expression of hypothalamic peptides in the pituitary. Six-week-old female rats were injected subcutaneously with DHT (5 mg/kg/day) daily for 7 days. After the rats were euthanized, the anterior pituitary was removed from nontreated (*n* = 4) and DHT-treated rats (*n* = 4), and then mRNA was extracted from the anterior pituitary tissue and reverse transcribed. Gnrh-r (a), Trh-r (b), Pac1-r (c), and Kiss1-r (d) mRNA levels were measured by quantitative RT-PCR. Samples from each experimental group were run in duplicate and normalized to the mRNA levels of the housekeeping gene Gapdh. The results are expressed as fold induction over the control and presented as the mean ± SEM. ^*∗∗*^*P* < 0.01, ^*∗*^*P* < 0.05 versus the control.

Synthesis and secretion of gonadotropins or prolactin are controlled not only by its principle stimulators, GnRH or thyrotropin-releasing hormone (TRH), but also by other hypothalamic peptides such as pituitary adenylate cyclase-activating polypeptide (PACAP) [25, 26] and kisspeptin [27, 28]. *Gnrh-r* gene expression in the anterior pituitary gland was significantly reduced in rats treated with DHT compared with those in nontreated rats (0.81 ± 0.004-fold) (Figure 3(a)). TRH receptor (*Trh-r*) gene and kisspeptin receptor (*Kiss1-r*) gene expression levels were unchanged by DHT (Figure 3(b) and 3(d)), but PACAP type I receptor (*Pac1-r*) gene expression was significantly increased in the anterior pituitary gland in rats treated with DHT (1.64 ± 0.29-fold) (Figure 3(c)).

### 3.4. Effect of DHT Administration on Gene Expression Levels of Inhibin Subunits and Follistatin within the Anterior Pituitary

**Figure 4:**
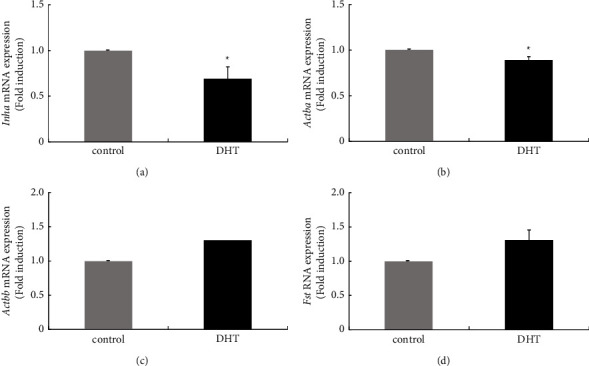
Effect of dihydrotestosterone (DHT) administration on the gene expression of inhibin/activin subunits and follistatin in the anterior pituitary. Six-week-old female rats were injected subcutaneously with DHT (5 mg/kg/day) daily for 7 days. After the rats were euthanized, the anterior pituitary was removed from nontreated (*n* = 4) and DHT-treated rats (*n* = 4), and then mRNA was extracted from the anterior pituitary tissue and reverse transcribed. Inha (a), Actba (b), Actbb (c), and Fst (d) mRNA levels were measured by quantitative RT-PCR. Samples from each experimental group were run in duplicate and normalized to the mRNA levels of Gapdh. The results are expressed as fold induction over the control and presented as the mean ± SEM. ^*∗*^*P* < 0.05 versus the control.

It is known that the activin, inhibin, and follistatin systems have roles in the regulation of pituitary hormones [29, 30]. mRNA expression levels of inhibin/activin subunits and follistatin in the anterior pituitary were compared between control and DHT-treated rats. Inhibin *α* subunit (*Inha)* gene expression in the anterior pituitary was significantly reduced in rats treated with DHT, with a decrease to 0.69 ± 0.13-fold (Figure 4(a)). Activin *β*A subunit gene (*Actba*) expression was also slightly reduced by DHT (0.89 ± 0.04-fold) (Figure 4(b)). In contrast, expression levels of activin *β*B subunit (*Actbb*) and follistatin (*Fst*) genes were unchanged by DHT treatment (Figure 4(c) and 4(d)).

#### 3.4.1. Effect of DHT on the Expression of GnRH-R, Pac1-R, and Inha- and *β*-subunits in Mouse Pituitary Gonadotroph L*β*T2 Cells

**Figure 5:**
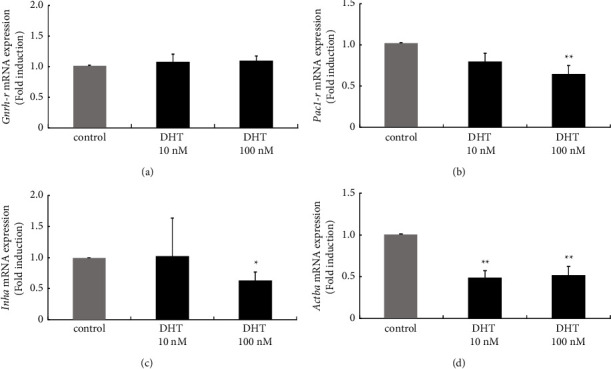
Effect of dihydrotestosterone (DHT) stimulation on the gene expression of GnRHR, PAC1R, and inhibin subunits in gonadotroph L*β*T2 cells. L*β*T2 cells were stimulated with the indicated concentrations of DHT for 24 h After stimulation, mRNA was extracted and reverse transcribed, and mRNA levels of Gnrh-r (a), Pac1-r (b), and Inha (c) and Actba were measured by quantitative RT-PCR. Samples from each experimental group were run in duplicate and normalized to the mRNA levels of the housekeeping gene Gapdh. The results are expressed as fold induction over unstimulated cells and presented as the mean ± SEM of three independent experiments. ^*∗∗*^*P* < 0.01, ^*∗*^*P* < 0.05 versus control.

*In vivo* experiments using female rats demonstrated that DHT exposure reduced *Gnrh-r* expression and increased *Pca1-r* expression in the anterior pituitary gland. To examine the effect of DHT on the population of gonadotrophs, mouse gonadotroph model L*β*T2 cells were stimulated with DHT and examined. Both 10 nM and 100 nM DHT treatments failed to modulate *Gnrh-r* gene expression in L*β*T2 cells (Figure 5(a)). *Pac1-r* gene expression in these cells was slightly inhibited by DHT stimulation, with a reduction to 0.65 ± 0.1-fold by 100 nM DHT stimulation (Figure 5(b)). Similar to the phenomenon observed in the *in vivo* experiment, expression levels of *Inha* and *Actba* genes in L*β*T2 cells were reduced by DHT stimulation and were reduced to 0.63 ± 0.14-fold and 0.52 ± 0.10-fold by 100 nM DHT stimulation, respectively (Figure 5(c) and 5(d)).

#### 3.4.2. Effect of DHT on Prl and Pac1-R Gene Expression in the GH3 Somatolactotroph Cell Model

**Figure 6:**
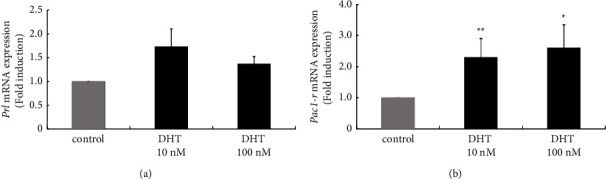
Effect of dihydrotestosterone (DHT) stimulation on Prl and Pac1-r gene expression in rat somatolactotroph GH3 cells. GH3 cells were stimulated with the indicated concentrations of DHT for 24 h After stimulation, mRNA was extracted and reverse transcribed, and Prl (a) and Pac1-r (b) mRNA levels were measured by quantitative RT-PCR. Samples from each experimental group were run in duplicate and normalized to the mRNA levels of the housekeeping gene Gapdh. The results are expressed as fold induction over unstimulated cells and presented as the mean ± SEM of three independent experiments. ^*∗∗*^*P* < 0.01, ^*∗*^*P* < 0.05 versus control.

In DHT-treated rats, *Prl* gene expression in the anterior pituitary was significantly decreased compared with control rats. In rat somatolactotroph GH3 cells, *Prl* gene expression was unchanged by both 10 nM and 100 nM DHT treatment (Figure 6(a)). In contrast, DHT stimulation significantly increased *Pac1-r* expression under 10 nM and 100 nM DHT treatment by 2.30 ± 0.60 and 2.61 ± 0.74-fold, respectively (Figure 6(b)). The effect of DHT on *Pac1-r* expression in GH3 cells was quite similar to that observed in the rat anterior pituitary *in vivo*.

#### 3.4.3. Effect of Activin-A and Inhibin-A on Gonadotropin Subunit Gene Expression in L*β*T2 Cells

**Figure 7:**
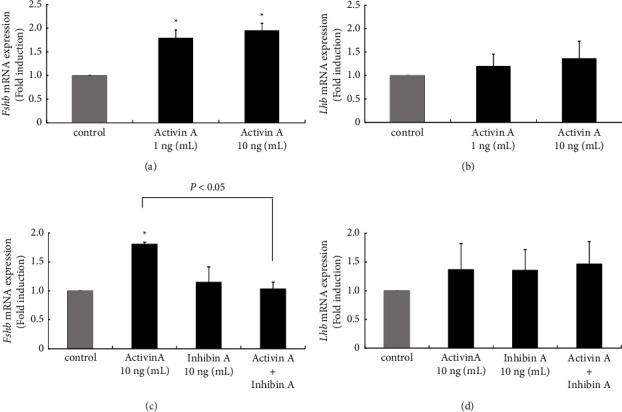
Effect of activin A and inhibin A on gene expression of gonadotropin subunits in gonadotroph L*β*T2 cells. L*β*T2 cells were stimulated with the indicated concentrations of activin (a) inhibin (b) or together with activin A and inhibin B for 24 h After stimulation, mRNA was extracted and reverse transcribed, and mRNA levels of Fshb (A, C) and Lhb (B, D) subunit genes were measured by quantitative RT-PCR. Samples from each experimental group were run in duplicate and normalized to the mRNA levels of the housekeeping gene Gapdh. The results are expressed as fold induction over unstimulated cells and are presented as the mean ± SEM of three independent experiments. ^*∗*^*P* < 0.05 versus the control. The difference between activin A and activin *A*+ inhibin A on Fshb mRNA was statistically different (*P* < 0.05).

The effect of activin A and inhibin A on gonadotropin subunit gene expression was examined using L*β*T2 cells because DHT treatment significantly reduced *Inha* and *Actba* subunit gene expression *in vivo*. Because dimers of activin *β*A compose activin A, whereas heterodimers of inhibin *α* and activin *β*A subunit compose inhibin A, we next tested the effect of activin A and inhibin A on gonadotropin-subunit gene expression using L*β*T2 gonadotroph cells. As expected, both 1 ng/mL and 10 ng/mL concentrations of activin A significantly increased *Fshb* gene expression, by 1.80 ± 0.17 and 1.96 ± 0.15-fold, respectively (Figure 7(a)). *Lhb* gene expression in L*β*T2 cells was unchanged by both concentrations of activin A stimulation*β* (Figure 7(b)). Inhibin-A itself did not modulate the gene expression of *Fshb* and *Lhb* gonadotropin subunits (Figures 7(c) and 7(d), but the activin A–induced increase in *Fshb* gene expression was inhibited in the presence of inhibin A (Figure 7(c)).

#### 3.4.4. Effect of Activin A and Inhibin A on Prolactin Gene Expression in GH3 Cells

**Figure 8:**
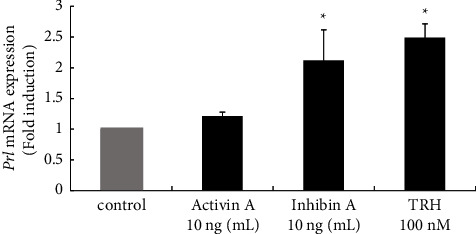
Effect of activin A and inhibin A on Prl gene expression in lactotroph GH3 cells. GH3 cells were stimulated with the indicated concentrations of activin A or inhibin A for 24 h After stimulation, mRNA was extracted and reverse transcribed, and Prl mRNA levels were measured by quantitative RT-PCR. Samples from each experimental group were run in duplicate and normalized to the mRNA levels of the housekeeping gene Gapdh. The results are expressed as fold induction over unstimulated cells and are presented as the mean ± SEM of three independent experiments. ^*∗*^*P* < 0.05 versus the control.

In prolactin-producing GH3 cells, activin A failed to stimulate *Prl* gene expression. In contrast, 10 ng/mL inhibin A stimulation significantly increased *Prl* mRNA expression, by 2.48 ± 0.42-fold compared with nonstimulated control cells. TRH stimulation was applied as a positive control (Figure 8).

## 4. Discussion

In this study, we examined the effects of 7 days of administration of exogenous androgen in ovary-intact female rats. Because the rats used in this experiment possessed a normal oestrogen milieu, our *in vivo* experiments reflect the endocrinological situation in reproductive women with hyperandrogenism. After induction of hyperandrogenism in rats by 7 days of administration of DHT, estrous cyclicity determined by vaginal smear was not drastically disrupted, and the expression levels of gonadotropin *Cga*, *Lhb*, and *Fshb* genes were unchanged; however, *Prl* gene expression was repressed in the pituitary gland. DHT administration not only influenced prolactin synthesis but also induced changes in gene expression of receptors for hypothalamic factors. Among receptors for GnRH, TRH, PACAP, and kisspeptin, *Gnrh-r* gene expression was decreased, and *Pac1-r* gene expression was increased within the pituitary gland by DHT treatment. Furthermore, in rat pituitary tissue, local expression of *Inha* and *Actba* subunit genes, which encode the components activin A and inhibin A, was decreased by DHT treatment.

It is well known that elevated serum levels of androgen disrupt gonadal function and lower gonadotropin levels. Anaabolic steroid abuse in healthy cisgender women and the taking of androgen supplements by transgender men both lower gonadotropin levels and suppress menstrual cycles [31, 32]. Indeed, a recent study by Esparza et al. clearly showed that 3 weeks of androgen supplementation reduced *in vivo* LH pulsatility, with decreases in pulse frequency, amplitude, peak, and basal secretion of LH in mice. They also revealed that androgen supplementation suppressed the expression of *Kiss-1* (which encodes kisspeptin) and *Tac2* (which encodes neurokinin B; NKB) in the kisspeptin neurons within the arcuate nucleus (ARC) region of the hypothalamus [33]. The neuronal population of kisspeptin neurons, called KNDy neurons, coexpress NKB and dynorphin A (Dyn A) [34], and they are a known component of GnRH pulse generator mechanisms [35, 36]. In addition, kisspeptin secretion is stimulated and inhibited by NKB and Dyn A, respectively [37]. Thus, in consideration of the previous report by Esparza et al. [33], hyperandrogenism might have some suppressive effect on NKB expression in KNDy neurons, thereby inhibiting kisspeptin release, resulting in a decrease in LH pulsatility.

Esparza et al. [33] examined the effect of DHT in ovariectomized (OVX) rats by observing the pattern of gonadotropin secretion and changes in Kiss1 and NKB staining in the ARC region of the hypothalamus by comparing them to non-DHT-treated OVX mice. OVX mice were artificially induced to have a high LH pulse frequency, which was caused by an elevation of kisspeptin in KNDy neurons and subsequently higher GnRH pulse secretion. In contrast, in our *in vivo* experiment, DHT was administered to ovary-intact female rats for only 7 days. Under this treatment, estrous cyclicity was still present in DHT-treated rats. Although it is unclear how DHT supplementation changed LH pulse frequency and amplitude in ovary-intact female rats, it is possible that the pattern of LH secretion was not drastically changed by 7 days of DHT treatment because estrous cyclicity was not disrupted. Indeed, in our assays focused on the anterior pituitary, *Cga*, *Lhb*, and *Fshb* gene expression levels within the pituitary were unchanged by DHT treatment. If the pattern of GnRH release, especially its pulse frequencies, were changed by DHT, the synthesis of gonadotropin subunit expression should have been changed because gonadotropin subunits, especially the expression of *Lhb* and *Fshb* genes, are specifically regulated by the pulsatile pattern of GnRH [38]. Thus, it is plausible that the pattern of release of GnRH is not dramatically altered in ovary-intact rats by 7 days of treatment with DHT or that alteration of the GnRH secretory pattern in our treatment does not have a significant impact on gonadotropin subunit synthesis in the pituitary gland. However, we have recently revealed that *Kiss-1* and *Tac2* gene expression levels in the ARC region of the hypothalamus were increased by 7 days of DHT administration in an ovary-intact rat model [21]. These results imply that hyperandrogenism in ovary-intact female rats could be the cause of increased activity of KNDy neurons and the subsequent increase in GnRH pulse frequencies. Osuka et al. demonstrated that female rats that were exposed to DHT prenatally and postnatally exhibited PCOS phenotypes characterized by higher LH secretion with higher kisspeptin and NKB levels in the ARC region of the hypothalamus [18]. If DHT is administered for longer periods, the expression patterns of gonadotropin subunits within the pituitary might be altered by the secretory pattern of kisspeptin and GnRH from the hypothalamus. In contrast, *Prl* gene expression in the anterior pituitary was repressed by 7 days of DHT treatment. We did not assess potential changes in the hypothalamic factors that regulate prolactin synthesis, such as TRH or dopamine. However, considering the *in vitro* observation that DHT did not modulate *Prl* gene expression in GH3 lactotroph cell lines, prolactin gene expression reduction in DHT-treated animals might be caused by other factors such as hypothalamic factors. In women, hyperandrogenemia and hyperprolactinemia are the most common etiologies of anovulation [12]. Previous reports demonstrated the suppressive effect of prolactin on KNDy neurons and the subsequent reduction in gonadotropin secretion [39, 40]. If kisspeptin/GnRH pulse secretion increases could be induced as a result of prolonged DHT treatment, DHT might strengthen their effect on KNDy neurons by preventing prolactin synthesis at the pituitary level.

In addition to the reduction of *Prl* gene expression, a reduction of *Gnrh-r* gene expression and an increase in *Pac1-r* gene expression were observed within the anterior pituitary of ovary-intact female rats after DHT treatment. Furthermore, local gene expression of the inhibin subunits that compose activin and inhibin was changed by DHT within the pituitary gland. These observations suggest that the anterior pituitary was functionally changed by 7 days of DHT stimulation. Although *Gnrh-r* gene expression was repressed by DHT, the expression levels of gonadotropin subunit genes were not altered, indicating that this change did not affect gonadotropin synthesis. However, although prolactin expression was repressed by DHT, *TRH-r* gene expression was not altered, indicating that the level of TRH receptor expression is not a major cause of the repression of prolactin expression. In this study, we also examined the expression levels of *Pac1-r* and *Kiss1-r* genes because both PACAP and kisspeptin can regulate gonadotropin and/or prolactin synthesis [25, 27, 28, 41]. *Pac1-r* gene expression was increased by DHT *in vivo*, but *Kiss1-r* gene expression was not modulated. However, unlike the phenomenon observed in the present *in vivo* experiments, DHT did not reduce *Gnrh-r* gene expression in L*β*T2 gonadotroph cells. Collectively, these results suggest that the observed reduction of *Gnrh-r* gene expression might not be a direct effect of DHT on gonadotrophs, but that it was instead mediated by some other hypothalamic factors. In addition, although DHT stimulation increased *Pac1-r* expression in the anterior pituitary cells of rats, it was repressed in L*β*T2 cells under a higher concentration of DHT. However, *Pac1-r* gene expression in GH3 cells was increased by DHT stimulation, resembling the results from *in vivo* experiments. The effect of DHT on *Pac1-r* gene expression might be cell-specific and completely responsible for the observed increase in *Pac1-r* gene expression in the pituitary gland *in vivo*. Of course, phenomena revealed by *in vivo* experiments are not always mirrored by phenomena revealed by *in vitro* study using cell models because various complementary systems can function *in vivo*. In addition, the characteristics of hormone-producing cell models might be altered from their original characteristics by immortalization or multiple passages. For example, unlike the prolactin-secreting cells *in vivo*, prolactin-secreting GH3 cells were already devoid of functional dopamine receptors [42]. In addition, we should always bear in mind the possibility of inconsistent experimental results due to unskilled experimental manipulation in each experiment. Accordingly, the results obtained from both *in vivo* and *in vitro* experiments should be carefully considered.

We also examined the inhibin subunits and follistatin in the pituitary gland because locally produced inhibin, activin, and follistatin play roles in the regulation of gonadotropin, especially FSH, within the pituitary gland [43, 44]. The *in vivo* experiments using female rats showed that DHT treatment significantly reduced expression of *Inha* and *Actba* subunit genes, whereas the expression of *Actbb* and *Fst* genes was unchanged. This phenomenon was also observed in L*β*T2 gonadotrophs, which constitutively express inhibin subunits and *Fst* genes. Activin consists of *β*-subunit heterodimers that are encoded by *Actba* and *Actbb* genes and produce activin A (*β*A/*β*A), B (*β*B/*β*B), and AB (*β*A/*β*B) [45]. In contrast, inhibin is a dimeric protein that consists of one inhibin *α* subunit and one of two inhibin *β* subunits, thus forming inhibin A (*α*/*β*A) or B (*α*/*β*B) [46]. Therefore, a reduction of *Inha* and *Actba* gene expression in the pituitary gland by DHT treatment indicates the possibility that the locally produced activin A and inhibin A are decreased within the pituitary gland after DHT treatment and have some effects on pituitary hormone synthesis. To indirectly demonstrate how reduction of locally produced activin A or inhibin A affects pituitary hormone synthesis, the direct effects of activin A and inhibin A on hormone-producing cell models were examined. In our experiment using L*β*T2 gonadotroph cells, activin A increased *Fshb* subunit expression but not *Lhb* expression, and these results were quite comparable to those of previous reports describing the specific effect of activin on FSH*β* subunit expression [29, 30]. These observations suggest that basal gene expression of *Fshb* within the pituitary gland might be reduced by the reduction of activin A. However, DHT administration, which might be repressed by activin A and inhibin A within the pituitary, did not reduce the basal levels of gonadotropin subunit gene expression in ovary-intact female rats, indicating that the reduction of activin A and inhibin A within the pituitary gland by DHT does not affect gonadotropin subunit gene expression *in vivo*. Although activin A had some effect on *Fshb* gene expression in the present *in vitro* experiments using gonadotroph cell models, GnRH might have been much more responsible for maintaining gonadotropin subunit gene expression in the pituitary gland *in vivo*. Within the pituitary gland, DHT treatment significantly repressed *Prl* expression in rats, and local activin A and inhibin A expression within the pituitary might be reduced under this condition. Although activin A did not modulate the expression of *Prl* gene expression in a GH3 lactotroph cell model, inhibin A significantly increased *Prl* gene expression. Based on these observations, we can speculate that reduced expression of inhibin A is responsible for decreasing prolactin gene expression in rats treated with DHT.

In this study, we examined changes in the expression levels of gonadotropin subunits, prolactin, receptors for hypothalamic peptides, and inhibin subunits in the anterior pituitary gland or pituitary cell models. The limitation of our current study is that changes in gene expression were determined only by quantitative RT-PCR analysis and we did not measure them at protein levels. Furthermore, the LH pulse frequency and amplitude after DHT treatment were not recorded in the *in vivo* study.

## 5. Conclusion

In this study, we examined the effects of 7 days of treatment with DHT on ovary-intact female rats. Under DHT treatment, basal gene expression levels of three gonadotropin subunits were unchanged, but *Prl* gene expression was repressed. DHT treatment induced a decrease in *Gnrh-r* expression and an increase in *Pac1-r* expression within the anterior pituitary. However, in the pituitary gonadotroph cell line L*β*T2, DHT stimulation did not modulate gene expression of *Gnrh-r* and instead decreased *Pac1-r* expression. In prolactin-producing GH3 cells, DHT did not repress *Prl* gene expression but did increase *Pac1-r* expression. Within the pituitary gland, DHT treatment induced repression of *Inha* and *Actba* subunit genes, which encode components of activin A and inhibin A. Hyperandrogenism induces various adverse effects in women of reproductive age. Our present observation indicates that the characteristics of pituitary hormone-secreting cells are also changed locally by hyperandrogenemia. Both central and local effects on hyperandrogenemia may disturb physical homeostasis and induce biological effects on the hormonal milieu.

## Figures and Tables

**Table 1:** Primer sequences used for measurement of gene expression using real-time quantitative PCR.

Gene	Forward	Reverse
Cga	CCAAAGATCCAGAGTTTGCAGG	AAGTGCTACAGTGGCAGTCC
Lhb	GCCGGCCTGTCAACGCAACC	GAGGGCCACAGGGAAGGAGA
Fshb	CCTAGGACCCAGCTACTGCT	CCTATCTGTCAGGACAGCGG
Prl	AATGACGGAAATAGATGATTG	CCAGTTATTAGTTGAAACAGA
Gnrh-r	CTAACAATGCGTCTCTTGA	TCCAGATAAGGTTAGAGTCG
Thr-rTAGCA	AGATGCTCGCAGTGGTTGTA	GGGCCACACTGTAGTTAGCA
Pac1-r	CTTGTACAGAAGCTGCAGTC	CCGGTGCTTGAAGTCCATAG
Kiss1-r	CTGCCACAGACGTCACTTTC	ACATACCAGCGGTCCACACT
Inha	GTGGGGAGGTCCTAGACAGA	GTGGGGATGGCCGGAATACA
Actba	GGAGTGGATGGCAAGGTCAACA	GTGGGCACACAGCATGACTTA
Actbb	GGTCCGCCTGTACTTCTTCGTCTT	GGTATGCCAGCCGCTACGTT
Fst	GTGACAATGCCACATACGCC	GCCTCTGCAGTTACGCAATAA

## Data Availability

The datasets used to support the findings of this study are available from the corresponding author upon request.
